# The Cell Adhesion Molecule “CAR” and Sialic Acid on Human Erythrocytes Influence Adenovirus *In Vivo* Biodistribution

**DOI:** 10.1371/journal.ppat.1000277

**Published:** 2009-01-02

**Authors:** Elena Seiradake, Daniel Henaff, Harald Wodrich, Olivier Billet, Matthieu Perreau, Claire Hippert, Franck Mennechet, Guy Schoehn, Hugues Lortat-Jacob, Hanna Dreja, Sandy Ibanes, Vasiliki Kalatzis, Jennifer P. Wang, Robert W. Finberg, Stephen Cusack, Eric J. Kremer

**Affiliations:** 1 European Molecular Biology Laboratory, Grenoble Outstation, Grenoble, France; 2 Institut de Génétique Moléculaire de Montpellier, CNRS 5535, Montpellier, France; 3 Universitiés Montpellier I & II, Montpellier, France; 4 Unit of Virus-Host Cell Interactions, UMR 5233, UJF-EMBL-CNRS, Grenoble, France; 5 Institut de Biologie Structurale, UMR 5075 CEA-CNRS-UJF, Grenoble, France; 6 Department of Medicine, University of Massachusetts Medical School, Worcester, Massachusetts, United States of America; Scripps Clinic and Research Foundation, United States of America

## Abstract

Although it has been known for 50 years that adenoviruses (Ads) interact with erythrocytes *ex vivo*, the molecular and structural basis for this interaction, which has been serendipitously exploited for diagnostic tests, is unknown. In this study, we characterized the interaction between erythrocytes and unrelated Ad serotypes, human 5 (HAd5) and 37 (HAd37), and canine 2 (CAV-2). While these serotypes agglutinate human erythrocytes, they use different receptors, have different tropisms and/or infect different species. Using molecular, biochemical, structural and transgenic animal-based analyses, we found that the primary erythrocyte interaction domain for HAd37 is its sialic acid binding site, while CAV-2 binding depends on at least three factors: electrostatic interactions, sialic acid binding and, unexpectedly, binding to the coxsackievirus and adenovirus receptor (CAR) on human erythrocytes. We show that the presence of CAR on erythrocytes leads to prolonged *in vivo* blood half-life and significantly reduced liver infection when a CAR-tropic Ad is injected intravenously. This study provides i) a molecular and structural rationale for Ad–erythrocyte interactions, ii) a basis to improve vector-mediated gene transfer and iii) a mechanism that may explain the biodistribution and pathogenic inconsistencies found between human and animal models.

## Author Summary

In most cases, adenoviruses are thought to initially enter the host via contact with epithelial cells and spread within the host via an unknown mechanism. Most adenovirus serotypes use a cell adhesion molecule dubbed “CAR” to attach to cells. To assess, predict and understand adenovirus biology and vectorology, many *in vivo* studies use mice and monkeys. These animal models have been considered reliable models in the realm of viral pathogenesis and gene transfer. One of the implications of our study suggests that the rat may be a more appropriate model during intravenous adenovirus delivery because like humans, and unlike mice and monkeys, they also express CAR on their erythrocytes. The identification of CAR on human erythrocytes explains a 50-year-old enigma of adenovirus hemagglutination, helps us better understand adenovirus *in vivo* biology and may open new avenues to understand the role of cell adhesion molecules during erythropoiesis.

## Introduction

Adenoviruses (Ads) are nonenveloped double-stranded DNA pathogens that infect all vertebrate classes. To date, all Ads display a characteristic, icosahedral symmetry in which 240 subunits of the trimeric hexon protein form the facets and 12 copies of the penton, comprising the pentameric penton base protein and the externally projecting trimeric fiber, form the vertices [1]. While the stoichiometry of the penton base and hexon is apparently conserved, the fiber can exist as a single or double copy at each vertex [2]. At least *in vitro* and for most cell types, the fiber mediates the initial attachment to primary receptors, such as the D1 domain of the coxsackievirus and adenovirus receptor (CAR), sialic acids, CD46, and others (for review see Zhang & Bergelson [3]). Interaction with auxiliary receptor(s), in particular some of the dimeric integrins via the Arg-Gly-Asp sequence (an integrin-interacting motif) on the penton base, may induce internalization of some serotypes. However, other auxiliary receptors or mechanism of internalization may exist for human serotypes 40 and 41 (HAd40/41), and canine serotype 2 (CAV-2), which have no identifiable integrin-interacting motif in the penton base [4],[5].

Greater than 150 Ad serotypes have been isolated. Approximately 50 of these are currently classed as human pathogens that, in most cases, generate subclinical ocular, respiratory and gastrointestinal tract infections. In the immunocompromised host however, lethal HAd infections can spread, via unknown mechanisms, to the kidney, liver and brain [6],[7]. The human Ads (HAds) are divided into subgroups (or species or subgenera) A - F. The triage of the human serotypes into subgroups is based in part on serotype-specific *ex vivo* erythrocyte cross-linking (or hemagglutination) [8]. The clinical hemagglutination assays use lysates from virus-infected cells to crosslink erythrocytes from a handful of species. This highly heterogeneous lysate contains whole virus particles, empty capsids, penton monomers, penton dodecahedrons, fiber monomers, hexon etc. By using fractionated infected cell lysate, a handful of laboratories found that the multivalent complexes containing fiber were responsible for hemagglutination [9]–[12].

Erythrocyte membranes contain highly sialated glycoproteins and glycolipids. One of the most abundant glycoproteins on erythrocytes is glycophorin A (∼10^5^ copies/cell). With its high sialic acid content, glycophorin A is the main contributor to the net negative cell-surface charge and is critical for minimizing cell–cell interactions and preventing erythrocyte aggregation [13]. Sialic acid is a collective term for a family of 9-carbon monosaccharides, which are often found as terminal sugar residues on glycans of glycoproteins and glycolipids (usually α2-3, -6 or -8 linked). In addition to some HAds, a number of viruses, including orthomyxoviruses, paramyxoviruses, picornaviruses, papovaviruses, coronaviruses, reoviruses and parvoviruses bind to sialic acids [14]–[18]. Possibly because erythrocytes from different species vary in their sialic acid content, the hemagglutination properties of sialic acid-binding viruses may also diverge [19].

HAd subgroup D (serotypes 9, 15, 19p and 37) and B:2 (serotypes 11a, 11p and 34a) erythrocyte binding depends on the fiber head and several attempts have been made to define the region(s) responsible [20]–[23]. Among the HAds, serotype 37 is unusual: its fiber head can bind CAR, CD46 and sialic acid, but the virus appears to use only the latter two as functional receptors [24]–[26]. Burmeister et al. found that the sialic acid moiety bound to a basic patch close to the center of the trimeric fiber heads of HAd37 and HAd19p [27]. Interestingly, the putative hemagglutination domain in subgroup D heads partially aligns with the sialyl-lactose binding site, which on the basis of sequence alignments is likely to be conserved in other members of this subgroup. These observations raise the question as to whether Ad-erythrocyte interaction is a consequence of fiber head-sialic acid interaction.

In this study we initially characterize the erythrocyte binding of unrelated serotypes, HAd5, HAd37 and CAV-2. Although both HAd37 and CAV-2 agglutinate human erythrocytes at low particle-to-cell ratios, they use different receptors (sialic acid and CD46 vs. CAR) [5],[25],[26], have different clinical tropisms (ocular vs. respiratory tract) and infect different species. We found that HAd37-erythrocyte interaction is primarily due to sialic acid binding. We show by structural analysis that the CAV-2 fiber head also contains a sialic acid binding site, but in contrast to HAd37 this site is modestly involved in hemagglutination. Unexpectedly, our biochemical and competition analyses suggested that CAV-2-erythrocyte interactions also depend on binding to CAR on human and rat erythrocytes. Using a transgenic mouse that expresses CAR on erythrocytes [28] we demonstrate that CAR-binding Ads can be sequestered by CAR-expressing erythrocytes, and prevent liver infection. Our study provides a molecular and structural rationale for the 50-year-old enigma of *ex vivo* Ad-erythrocyte interactions. In addition to the relevance for Ad pathogenesis and vector biology, the expression of CAR by human erythrocytes may shed light on the role of cell adhesion molecules during erythropoiesis.

## Results

### Hemagglutination assays using Ads

**Figure 1:**
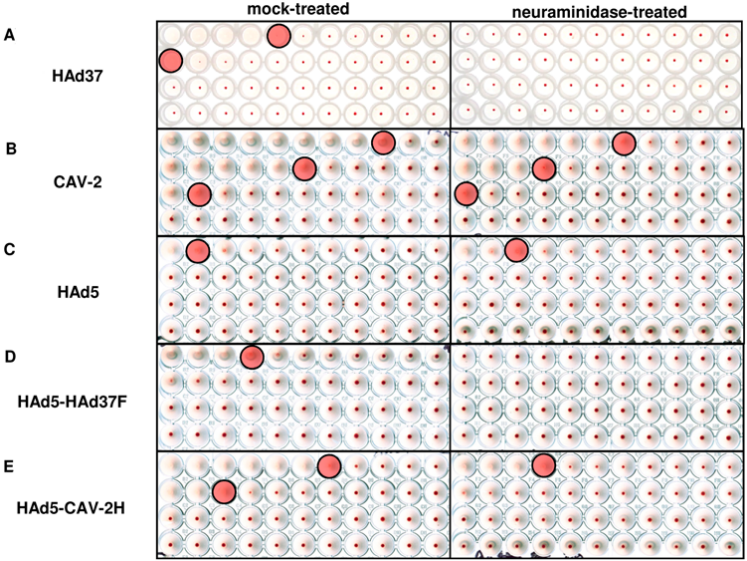
Hemagglutination assays with HAd37, CAV-2, HAd5 or hybrid capsids. Mock-treated (left hand column) or neuraminidase-treated (right hand column) erythrocytes (all wells contain the same number of cells) were incubated with serial dilutions (10-fold vertically and 2-fold horizontally) of A. HAd37; B. CAV-2; C. HAd5; D. HAd5-HAd37F, a hybrid capsid containing the HAd37 fiber on the HAd5 capsid; and E. HAd5-CAV-2H, a hybrid capsid containing the CAV-2 fiber head on the HAd5 capsid. Agglutination is visualized by the lack of erythrocyte sedimentation (no red spot) at the bottom of the conical well (see Figure S4 for graphic description). The pink shaded well is the first well that does not significantly agglutinate. All assays were performed at least three times.

Consistent with previous reports, we found that at a modest physical particle (pp)-to-erythrocyte ratio (∼130) HAd37 efficiently agglutinates human erythrocytes (Figure 1A). When compared to HAd37, CAV-2 hemagglutinates at ∼20-fold lower ratio (i.e. more efficiently, Figure 1B). In our hands, and consistent with others [29], HAd5 also agglutinated human erythrocytes, but at a higher ratio (>800 pp/erythrocyte) (Figure 1C). To assay the roles of HAd37 and CAV-2 capsid proteins, we incubated erythrocytes with chimeric HAd5 vectors harboring the fiber from HAd37 [30] or the fiber head from CAV-2 [31]. We found that the HAd5-HAd37F and HAd5-CAV-2H hybrid capsids induced agglutination at lower ratios than HAd5 but not quite to those of HAd37 and CAV-2 (Figure 1D and E). Pre-incubating erythrocytes with neuraminidase, which removes sialic acid from their membranes, eliminated agglutination by HAd37 and a HAd5-HAd37F hybrid capsid (Figure 1, right hand column). In contrast, removing sialic acid from erythrocyte membranes only modestly reduced CAV-2 and the hybrid HAd5-CAV-2H agglutination (data summarized in Table 1).

**Table 1:** Virus-induced hemagglutination

Hemagglutinin	Mock-treated	Neuraminidase-treated
CAV-2	7	20
HAd37	130	∞
HAd5	700	600
HAd5-CAV-2H	33	200
HAd5-HAd37F	200	∞
CAV-2 (GATA1-CAR erythrocytes)	10	10

The agglutination titer in physical particles-to-erythrocyte (1.5×10^6^ erythrocytes/well).

High numbers = poor agglutinator.

∞ = no agglutination

Together, these data suggest that the CAV-2 fiber head, like in subgroup D and B:2 HAds, is responsible for hemagglutination and that sialic acid binding plays a more significant role in HAd37 binding of human erythrocytes than it does for CAV-2.

To mimic the hemagglutination caused by HAd37 fiber head in a virion-like context, we generated a multivalent protein complex similar to the “complete hemagglutinin” described for HAd9 by Norrby et al. [9]. For this purpose HAd3 penton dodecahedra were incubated with chimeric “mini fibers” consisting of HAd3 fiber tail and shaft motifs 1+2 and HAd37 fiber head. In addition, we generated HAd37 fiber heads containing mutations in the sialic acid binding site [27] (see Table 2 for a list of fiber head mutants). The HAd3 penton dodecahedra without a fiber did not cause hemagglutination (data not shown). The dodecahedra containing a wild type HAd37 fiber head (HAd37H^wt^) (Figure S1) agglutinated erythrocytes at low protein concentrations (about 10^4^-fold less mass than HAd37 virions) (Table 2). Similar to HAd37, this “complete hemagglutinin” poorly agglutinates neuraminidase-treated erythrocytes (Table 2). The dodecahedra containing a HAd37 fiber with a Lys to Glu mutation at amino acid 345 in the sialic acid binding site (HAd37H^SA−1^), had at least a 10^5^-fold reduced hemagglutination activity compared to the dodecahedra containing HAd37H^wt^. The chimeric wild type CAV-2 fiber head (CAV-2H^wt^) did not bind to HAd3 penton dodecahedra, which precluded equivalent experiments.

**Table 2:** Relative hemagglutination titers of capsid subunits (higher numbers = poor agglutinator)

HAd3 dodecahedra	∞	∞
HAd3 dodecahedra with mini HAd37 head	10	1×10^6^
HAd3 dodecahedra with mini HAd37H^SA−1^	1×10^6^	∞
HAd37H^wt^: wild type HAd37 fiber head	10	∞
HAd37H^SA−1^: HAd37 fiber head with K345E mutation	∞	∞
HAd37H^SA−2^: HAd37 fiber head with Y312A mutation	∞	∞
HAd37H^CAR−1^: HAd37 fiber head with E351A mutation	10	∞
HAd37H^CAR−2^: HAd37 fiber head with S299A mutation	10	∞
CAV-2H^wt^: wild type CAV-2 fiber head	∞	∞
CAV-2H^SA−1^: CAV-2 fiber head mutant R515A	∞	∞
CAV-2H^SA−2^: CAV-2H^SA−1^ with a N435A mutation	∞	∞
CAV-2H^CAR−^: CAV-2 fiber head with E384A mutation (CAR binding site)	∞	∞
CAV-2H^wt^: trimeric clusters	1	∞
CAV-2H^SA−1^: trimeric clusters	1000	∞
CAV-2H^SA−2^: trimeric clusters	1000	∞

A relative comparison of protein concentrations that induced agglutination of human erythrocytes.

Higher numbers = poor agglutinator: Like sample (e.g. dodecahedra vs. dodecahedra, fiber heads vs. fiber heads, or trimeric clusters vs. trimeric clusters) can be compare.

The highest concentration of HAd3 dodecahedra alone or in complex with mini-fibers was 3×10^−4^ µg/µl.

The highest concentration of His-tagged CAV-2 fiber head constructs was 5×10^−4^ µg/µl. α(2-3,-6,-8 and -9) neuraminidase was used in this assay.

∞ = no agglutination.

These data suggest that the HAd37 fiber shaft, hexon and pIX do not play key roles during binding and that the sialic acid binding site is involved in agglutination of human erythrocytes.

### Binding of the fiber heads to erythrocytes and glycophorin

**Figure 2:**
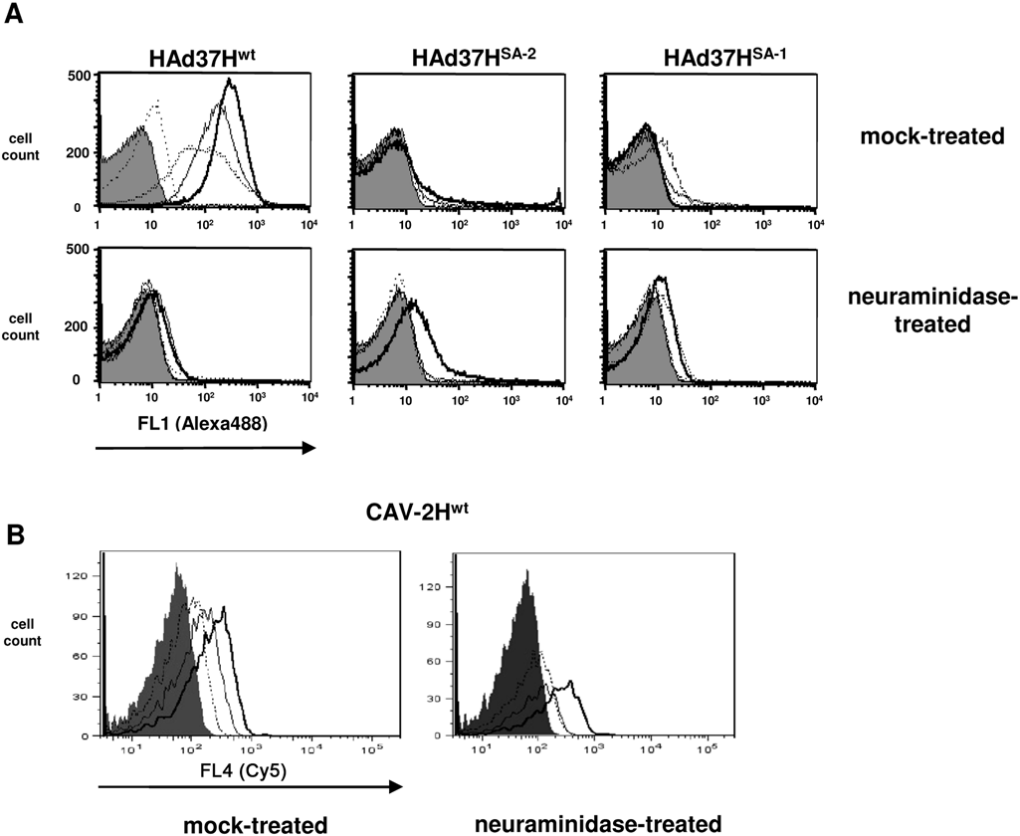
Binding of wild type and mutant fiber heads to human erythrocytes. A. HAd37 fiber head binding to erythrocytes (mock-treated, top panels or neuraminidase-treated, bottom panels) as measured by flow cytometry. The shadowed profile = mock-treated erythrocytes without fiber heads. The line graphs correspond to four 10-fold dilutions (13, 1.3, 0.13 and 0.013 mM) of three HAd37 fiber heads: HAd37^wt^ = wild type, HAd37^SA−1^ and HAd37^SA−2^ contain mutations in the HAd37H sialic acid binding domain. The lowest dilution (0.013 mM = the dotted line in the top left panel. The darkest line (furthest to the right in the top left panel) = 13 mM. Except for the modest binding of 13 mM HAd37^SA−2^ to neuraminidase-treated cells (bottom row middle panel), no other notable binding could be detected and therefore the line profiles overlap or are hidden by the shadowed mock-treated profile. B. Fluorescently-labeled CAV-2 fiber head (CAV2H^wt^) binding to mock-treated (left) or neuraminidase-treated (right) erythrocytes as measured by flow cytometry. Three 10-fold dilutions of CAV2H^wt^ (13, 1.3 and 0.13 mM) covalently labeled with Cy5 were assayed. The shadowed profile = mock-treated erythrocytes without fiber heads. As above the highest concentration of fiber head is in the darkest line profile. All assays were performed at least twice in triplicate.

To assay the binding of recombinant fiber heads, we incubated HAd37H^wt^ and mutant fiber heads with erythrocytes and then added anti-fiber head antibodies or antiserum. We then quantified attachment by flow cytometry. HAd37H^wt^ (Figure 2A) and mutants carrying point mutations in the CAR binding site (HAd37H^CAR−1^ and HAd37H^CAR−2^, mutation in Glu351 and Ser299, data not shown) [24] bound to erythrocytes in a dose-dependent manner. However, HAd37H^SA−1^ and HAd37H^SA−2^ (a Tyr to Ala mutation at amino acid 312), which harbor mutations in the sialic acid binding site, poorly bound erythrocytes (Figure 2A). In addition, all of the HAd37 fiber heads poorly bound neuraminidase-treated erythrocytes (Figure 2A, bottom row and data not shown).

Together, these data suggest that the HAd37 fiber head sialic acid binding site is a key determinant in the attachment to human erythrocytes.

Using the above approach, we detected minimal binding of CAV-2H^wt^ to erythrocytes (data not shown), possibly because the affinity of single CAV-2 fiber heads is low, the high affinity polyclonal antibody binding out-competed the weaker erythrocyte binding, and/or the number of CAV-2 fiber head receptors on erythrocytes is low. To address the potential competition, we incubated fluorescently labeled CAV-2 fiber heads with erythrocytes to circumvent the use of antibodies. Here we detected low, but reproducible, binding of CAV-2H^wt^ to mock-, as well as neuraminidase-treated, erythrocytes (Figure 2B).

Together, these data suggest that CAV-2 erythrocyte binding is notably less dependent on sialic acid than HAd37, and the lower binding may be due to reduced affinity or fewer receptors.

To address sialic acid-fiber head interaction using a cell-free system, glycophorin or asialoglycophorin (asialoGP) were immobilized on BIAcore sensor chips and increasing concentrations of HAd37 fiber heads were assayed. All binding curves had a square shape, suggesting high on and off rates (Figure S2). HAd37H^wt^ bound to glycophorin, but less efficiently to asialoGP. Compared to HAd37H^wt^, HAd37H^SA−2^ bound equally to asialoGP, but less to glycophorin. Interestingly, HAd37H^SA−1^ (Lys345Glu) bound less efficiently to glycophorin and asialoGP compared to HAd37H^wt^ and HAd37H^SA−2^, which suggests that HAd37 agglutination may be charge-dependent.

Consistent with the erythrocyte binding data, we found very little binding between CAV-2H^wt^ and glycophorin or asialoGP (data not shown). Overall, the SPR results reflect the results obtained using flow cytometry with mock or neuraminidase-treated erythrocytes.

### Structural analysis of the CAV-2 fiber head suggests a sialic acid binding site

Our results suggested that HAd37 hemagglutination was due to sialic acid binding via the sialic acid binding site in the fiber head. Although we have no evidence suggesting that CAV-2 can use sialic acid as a functional receptor, it is possible that the efficient CAV-2 agglutination of human erythrocytes is due to multiple and coordinated binding of sialic acid. For example, each of the 12 homotrimeric fiber heads on the end of the flexible CAV-2 shaft [32] could bind three sialic acid moieties (theoretically up to 36/capsid). Because the predicted subgroup D fiber head hemagglutination domains (in the CD and GH loops) appear to be well conserved, we tried to identify the corresponding domain in the CAV-2 fiber head using a mutagenesis strategy based on sequence homology (Figure S3). This approach was unsuccessful (data not shown), suggesting that Ad hemagglutination sites are not strictly conserved.

To address possible sialic acid binding via an alternative approach, we soaked CAV-2 fiber head crystals in solutions containing 2-3 sialyl-D-lactose. The crystal diffracted to 1.9 Å and the structure was solved by molecular replacement using our model of CAV-2 fiber head [24] (crystallographic details are summarized in Table S1). The electron density maps showed clear density for six N-acetyl neuraminic acid (Neu5AC) moieties in the asymmetric unit, three per fiber head trimer. Sialyl-lactose is composed of three sugar rings, the sialic acid moiety being linked to lactose (galactose-glucose). The density for the lactose moieties was weak, implying that these are flexible within the crystal. Only one of the six galactose rings in the asymmetric unit could be modeled. This molecule is located in between the two fiber head trimers in the asymmetric unit and forms a distant hydrogen bond (distance 3.25 Å) to Lys503 via the galactose oxygen 6. It is unlikely that this interaction is enough to immobilize the galactose ring because the other five galactose moieties in the asymmetric unit are not equally well visible in the electron density. More likely, the stabilization is due to the spatial restrictions imposed by the proximity of the other fiber head trimer.

**Figure 3:**
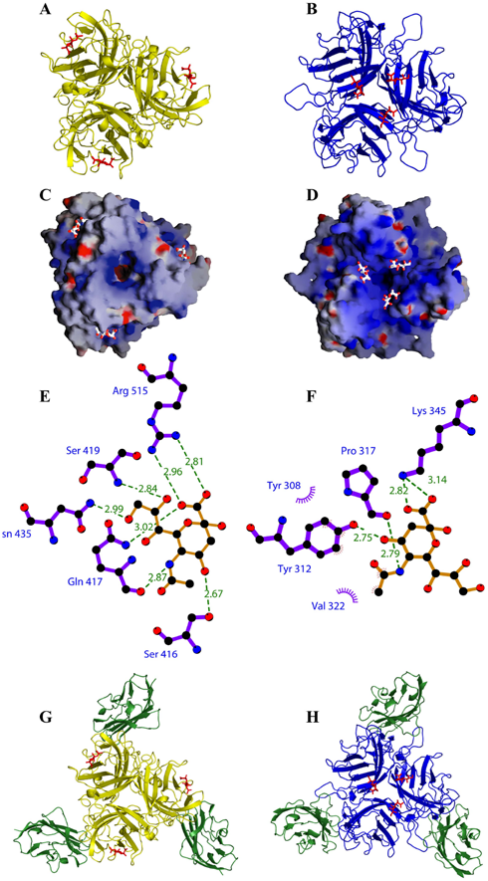
Structure of the CAV-2 fiber head in complex with sialic acid and CAR. A. Ribbon diagram of CAV-2 fiber head in complex with sialyl-D-lactose (red = Neu5AC, yellow = fiber head). B. Cartoon representation of HAd37 fiber head in complex with sialyl-D-lactose (red = sialic acid, blue = fiber head). C. Surface charge representation of CAV-2 fiber head and cartoon representation of sialic acid as found in the complex. D. Surface charge representation of HAd37 fiber head and cartoon representation of sialic acid as found in the complex. E. Representation of the contacts between CAV-2 fiber head (purple) and sialic acid (yellow). F. Representation of the contacts between HAd37 fiber head (purple) and Neu5AC (yellow). G. Ribbon diagram of CAV-2 fiber head in complex with CAR D1 and sialyl-D-lactose (red = Neu5AC, green = CAR, yellow = fiber head). H. Ribbon diagram of HAd37 fiber head in complex with CAR and sialyl-D-lactose (red = Neu5AC, green = CAR, blue = fiber head).

We found that the sialic acid binding site on CAV-2 head is distinct, both in sequence and location, from that found on the HAd37 fiber head. On the CAV-2 fibers head, sialic acid binds further away from the three-fold symmetry axis at the periphery of the trimer (Figure 3A–D). The residues involved in binding (Asn435, Ser419, Ser416, Gln417 and Arg515) do not align with those involved in sialic acid-binding by HAd37 fiber head (Figure S2). The HAd37 sialic acid binding site consists of three residues forming hydrogen bonds (Tyr213, Pro317 and Lys345) and two residues contacting sialic acid in hydrophobic interactions (Tyr308 and Val322) (Figure 3E). All seven interactions between CAV-2 fiber head and sialic acid are hydrogen bonds or salt bridges (Figure 3F), suggesting a relatively strong interaction compared to HAd37. Unlike HAd37 fiber head [33], no hydrophobic contacts contribute to sialic acid binding. Finally, sialic acid binds within a basic patch in each head (Figure 3C and D).

### CAV-2 and HAd37 fiber heads bind CAR and sialic acid simultaneously

Our results showing that the CAV-2 fiber head contains a sialic acid binding site creates a paradox: both HAd37 and CAV-2 fiber heads contain CAR and sialic acid binding sites - but HAd37 uses sialic acid and CD46, while CAV-2 uses CAR (CAV-2 does not use CD46 to infect cells, unpublished data) to infect cells. To determine if sialic acid and CAR binding were mutually exclusive, we soaked CAV-2 or HAd37 fiber head crystals in complex with CAR in solutions containing sialyl-D-lactose. Crystals containing the complex with CAV-2 fiber head diffracted to 2.9 Å and contained 12 chains of fiber head bound to 12 chains of CAR D1 and 12 sialyl-D-lactose molecules in the asymmetric unit (space group I422). Crystals containing the complex with HAd37 fiber head diffracted to 1.55 Å and contained one chain of fiber head in complex with one CAR D1 molecule and one sialyl-D-lactose molecule in the asymmetric unit (space group I23). For both structures the electron density maps showed clear density for sialic acid, but not lactose, at the expected positions on the fiber heads (Figure 3G and H).

Together, our data demonstrate that CAV-2 and HAd37 sialic acid binding sites do not overlap with the CAR binding sites, and both fiber heads can bind CAR and sialyl-D-lactose simultaneously.

### CAV-2 fiber head-erythrocyte interactions may be partially charge dependent

**Figure 4:**
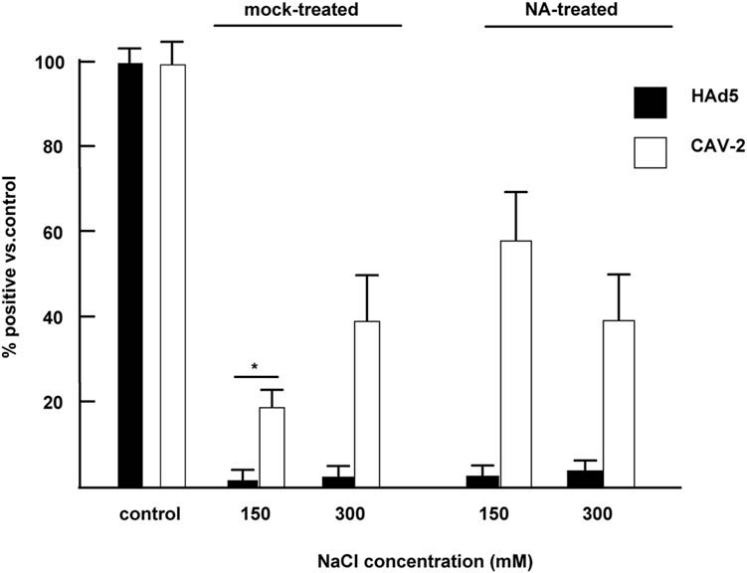
HAd5 and CAV-2 binding to human erythrocyte. CAVGFP or AdGFP was incubated with 2.5×10^7^ erythrocytes at 1 particle per cell (equivalent to ∼5×10^9^ pp in 1 ml of blood) in PBS and ± additional NaCl. The erythrocytes were pelleted by slow speed centrifugation, and an aliquot of the supernatant was removed and incubated with a monolayer of the most permissive and sensitive cells (911 cells for AdGFP and DKCre cells for CAVGFP). These latter cells were analyzed for GFP expression by flow cytometry 24 hr post-incubation. The percent of GFP^+^ cells versus the control is shown for mock- or neuraminidase-treated erythrocytes. All samples are significantly (*P*<0.05) different from the controls. In addition, the star (*) corresponds to a *P* value of <0.01 between the mock-treated erythrocytes at physiologically relevant salt concentration (150 mM) and the controls.

Arnberg and colleagues previously showed that HAd37 interaction with sialic acid was inhibited at high salt concentration [33]. To determine if a CAV-2-erythrocyte interaction was at least partially charge dependent, we incubated a CAV-2 vector expressing GFP (CAVGFP) or a HAd5 vector expressing GFP (AdGFP) with mock- or neuraminidase-treated erythrocytes in PBS containing increasing concentrations of ions. We then pelleted the erythrocytes by centrifugation, removed an aliquot of the supernatant, added it to cells, and assayed the cells for GFP expression by flow cytometry 24 hr post-infection. Using mock-treated erythrocytes (Figure 4), we found that at physiological salt concentrations ∼80% of CAVGFP was removed from the supernatant, while at higher salt concentration (300 mM NaCl) ∼60% of CAVGFP was removed. When using neuraminidase-treated erythrocytes, we again found that CAVGFP was efficiently removed from the supernatant. All the test samples were significantly (*P*<0.01) different from the control, as well as from the mock-treated 150 mM NaCl (*P*<0.05). Consistent with other studies [29],[34] AdGFP was also efficiently (90%) removed from the supernatant after the incubation with mock- (or neuraminidase-) treated erythrocytes.

These data suggest that, like HAd37, a modest degree of CAV-2-erythrocyte interactions may depend on electrostatic interactions. In addition, both the CAR-tropic adenoviruses (HAd5 and CAV-2) bind human erythrocytes at physiological salt concentrations.

### CAR expression by erythrocytes influences CAV-2 agglutination

While our results show that HAd37 hemagglutination is due primarily to sialic acid binding, CAV-2 hemagglutination appeared more complex. We therefore developed additional tests to address the molecular and structural basis. Due its sensitive and semi-quantitative potential, we returned to hemagglutination assays to understand the erythrocyte interactions.

Freshly purified CAV-2H^wt^, which predominantly consists of individual trimeric fiber heads, did not cause hemagglutination, presumably because it is not sufficiently multivalent to crosslink erythrocytes. However, several His-tagged Ad fiber heads, including those of HAd41 short fiber [35] and HAd37, form multimers that dissociate into single trimeric fiber heads upon removal of the histidine tag. Similarly, His-tagged CAV-2 fiber head forms multimers of a defined size that are stable on a size-exclusion column and can be visualized with an electron microscope (not shown). Removal of the His-tag yields single fiber heads. We found that His-tagged CAV-2H^wt^ agglutinated erythrocytes, while His-tagged CAV-2H^SA−1^ and CAV-2H^SA−2^ (two CAV-2 fiber heads with one or two mutations in the sialic acid binding site, see Table 2) showed reduced hemagglutination titers. However, we could not exclude the possibility that the reduced hemagglutination in these latter CAV-2H constructs was due to electrostatic interactions (the mutations modified the charge of the fiber heads).

**Figure 5:**
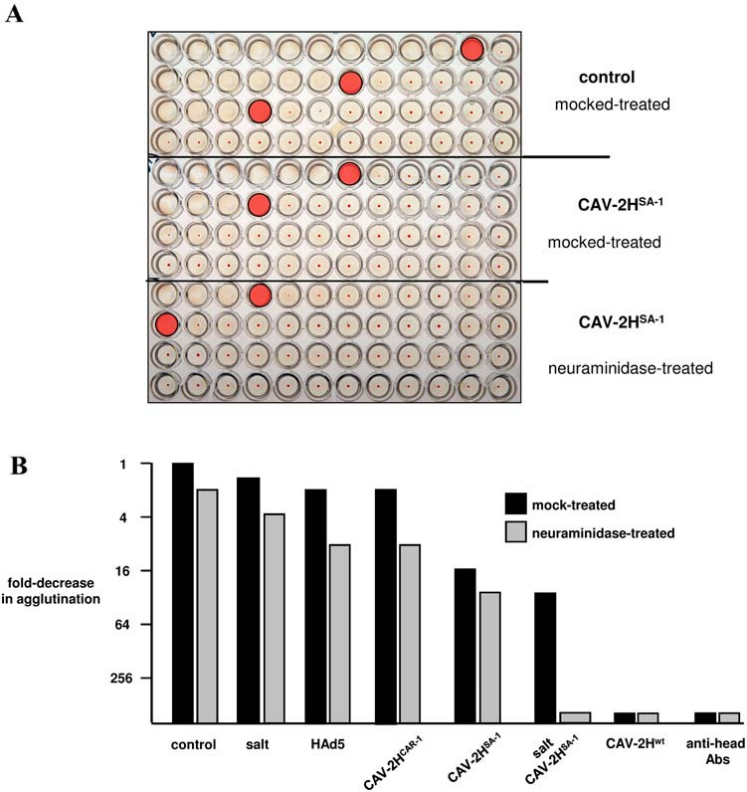
CAV-2 hemagglutination assays using competition with fiber heads, ± increased salt concentrations and ± neuraminidase. A. Examples of the results from competition assay (see Figure S4 for the schema of the approach). The pink shaded well corresponds to the last well that shows agglutination. A shift to the left of the pink shade signifies decreased agglutination due to blocking of the virus binding site or removal of an attachment ligand. A 2-fold decrease in agglutination would correspond to a one-well shift to the left of the last well that showed agglutination. Top control panel = agglutination of human erythrocytes by CAV-2 (for example ∼8 pp/erythrocyte in the pink shaded well on top row). Middle panel = erythrocytes preincubated with CAV-2H^SA−1^ (the CAV-2 fiber head that contains a mutation in the sialic acid binding site but can still bind CAR) prior to the agglutination assay (∼96 pp/erythrocyte in the pink shaded well, top row), which equals an ∼8 to 16-fold decrease from the control). Bottom panel = the same as the middle panel except that the erythrocytes were pretreated with neuraminidase (16 to 32-fold decrease). B. The graph shows the cumulative data from competition assays. CAV-2- agglutination of mock-treated erythrocytes was used as the starting point. The blocking agent was used with mock (black bars) or neuraminidase-treated (grey bars) erythrocytes. All assays were performed at least three times.

We next assayed CAV-2 hemagglutination using a competition assay (see Figure S4 for schema). In these competition assays, we pre-incubated erythrocytes with fiber heads, antibodies, salt and/or neuraminidase. Then the erythrocytes were incubated with CAV-2 and compared to mock-treated erythrocytes. We found that hemagglutination could be >256-fold reduced by pre-incubating CAV-2 with anti-fiber head antibodies, or pre-incubating erythrocytes with CAV-2H^wt^ (see Figure 5A for specific example and 5B for cumulative data). Pre-incubating the erythrocytes with the head from HAd5 or performing the assay in 225 mM NaCl led to modest 2 to 4-fold reductions. Unexpectedly, we found that like CAV-2H^wt^, CAV-2H^SA−1^ notably reduced agglutination (∼16-fold), while a CAV-2 fiber head with a mutation in the CAR binding site (CAV-2H^CAR−^) had a modest ∼2-fold reduction. In most cases, pre-treating erythrocytes with neuraminidase had a fairly small additive effect of CAV-2 hemagglutination.

Together, these data suggest that the CAR binding site, which is present in CAV-2H^wt^ and CAV-2H^SA−1^ but not CAV-2H^CAR−^, is involved in CAV-2 agglutination of human erythrocytes. That the CAV-2 CAR binding site is involved in erythrocyte binding is consistent with the study by Nicol et al. [36], which showed that a CAR-ablated HAd5 vector no longer agglutinated human and rat erythrocytes.

**Figure 6:**
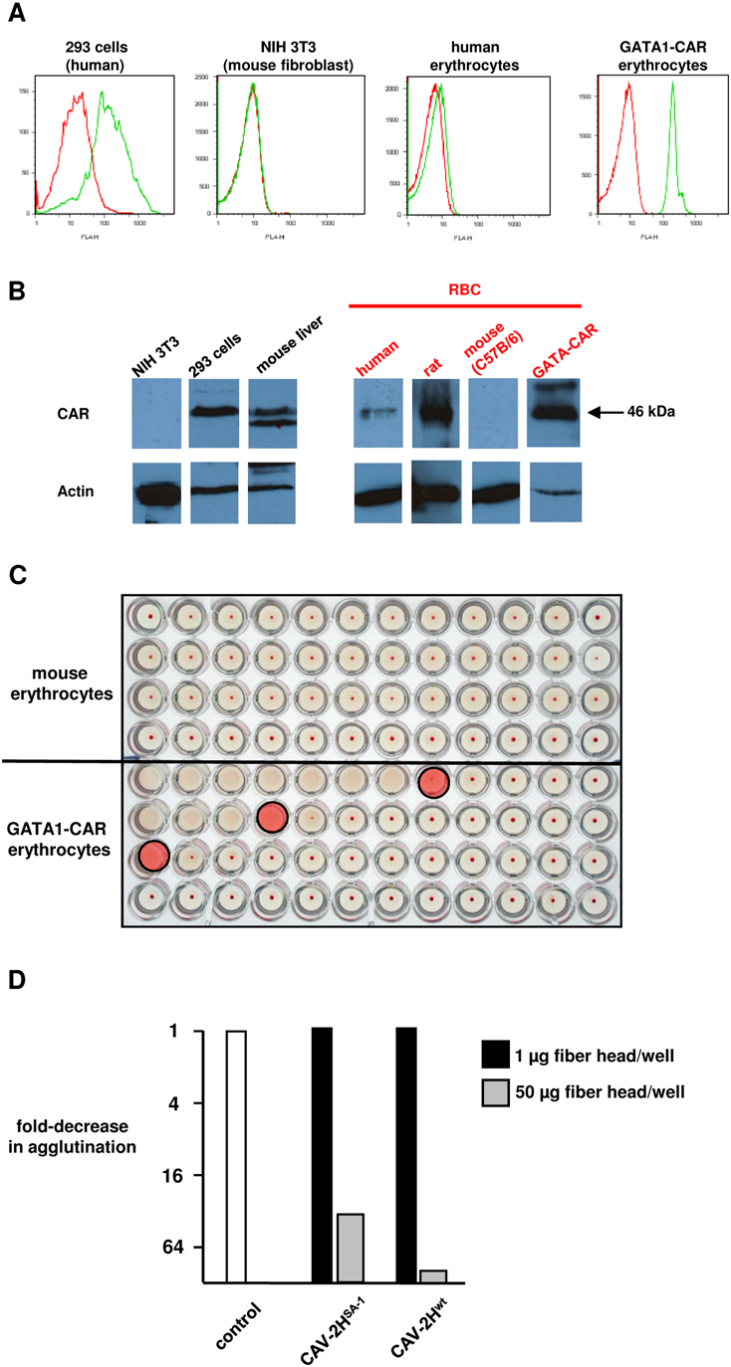
CAR expression by erythrocytes leads to CAV-2 induced agglutination. A. Flow cytometry analysis of CAR expression by human, mouse and GATA1-CAR mouse erythrocytes using the anti-CAR E1.1, which recognizes the native form of CAR. B. Western blot analysis of CAR expression using the polyclonal anti-CAR antibody CAR1605, which recognizes the denatured form of CAR: murine fibroblast (NIH 3T3 cells, CAR^−^), human embryonic kidney cells (293 cells, CAR^+^) and mouse liver (CAR^+^), and human, rat, mouse (C57BL/6) and GATA1-CAR mouse erythrocytes. An anti-actin staining was used to control the protein input. C. Hemagglutination assay using C57BL/6 and GATA1-CAR mouse erythrocytes. No significant agglutination was found with C57BL/6 mice while GATA1-CAR erythrocytes agglutinated with ∼3 pp of CAV-2/erythrocyte (pink shaded wells). D. Hemagglutination competition assay using GATA-CAR erythrocytes and performed as described in Figure 5 using a higher concentration of the fiber head (as would be expected due to the higher levels of CAR on GATA-CAR erythrocytes).

The presence of CAR on erythrocytes would be inconsistent with other reports [34]. Among other functions, CAR acts as a homodimeric cell adhesion molecule at tight gap junctions [37]. However, the expression of cell adhesion molecules during erythropoiesis is not unprecedented [38]. Erythrocytes from some species express cell adhesion molecules during the early stages of differentiation that are thought to be involved in interaction with macrophages. We therefore incubated erythrocytes with anti-CAR antibodies that recognize the extracellular domain of CAR and assayed expression using flow cytometry. We found low, but reproducible, CAR expression on the cell surface of human erythrocytes (Figure 6A). To assay CAR expression using another approach, we used western blot analysis to screen erythrocytes from several species. Using an anti-CAR Ab that recognizes the cytoplasmic domain of CAR we found CAR expression on human and rat, but not on mouse, dog, rabbit and nonhuman primate erythrocytes (Figure 6B and data not shown). Again, our results showed a relatively low level of CAR on human erythrocytes, which is consistent with the low level of CAV-2H^wt^ binding to erythrocytes (Figure 2B).

These data suggest that CAR binding is a significant factor in CAV-2, and likely other CAR-tropic Ads, interaction with human erythrocytes. This also is consistent with the fact that CAV-2 agglutinates rat erythrocytes, but not erythrocytes from mice, dogs, rabbits or some nonhuman primates (as well as other species) (Figure S5).

If CAR binding plays a role in CAV-2 agglutination, then knocking down/blocking or artificially expressing CAR on erythrocytes should prevent/induce hemagglutination. Eliminating CAR on mature enucleated erythrocytes is technically challenging. Furthermore, to the best of our knowledge there are no known anti-CAR antibodies that completely block Ad attachment. On the other hand, CAR has been expressed on erythrocytes using a transgenic mouse line (GATA1-CAR) with the CAR cDNA downstream of the globin transcription factor 1 promoter [28]. Using flow cytometry and western blot analysis (Figure 6A & B), we compared the levels of human CAR expressed by GATA1-CAR and human erythrocytes. We then repeated the hemagglutination assays using erythrocytes from control (C57BL/6) and GATA1-CAR mice. We also found that CAV-2 agglutinated GATA1-CAR erythrocytes at a low particle-to-cell ratio (Figure 6C), while there was no agglutination of C57BL/6 erythrocytes. Equally important, competition assays with recombinant CAV-2 fiber heads gave profiles that were similar to when we used human erythrocytes (Figure 6D, and data not shown).

Together, our data demonstrate that CAR expression by erythrocytes can lead to agglutination by CAR-tropic adenoviruses.

### The role of CAR in Ad biodistribution and pathogenesis

In spite of recent notable advances [39],[40], *in vivo* Ad biodistribution, tropism and pathogenesis for the CAR-tropic HAds are still poorly understood. Group C HAd serotypes 2 and 5 are the prototype Ads in terms of structure, tropism and pathogenesis. However, tropism has been primarily studied *in vitro, ex vivo* or in animal models. Our results showing that human and rat erythrocytes harbor CAR on their external membranes while mice and nonhuman primates do not, suggests that these latter animals poorly mimic the *in vivo* environment that Ads encounter.

**Figure 7:**
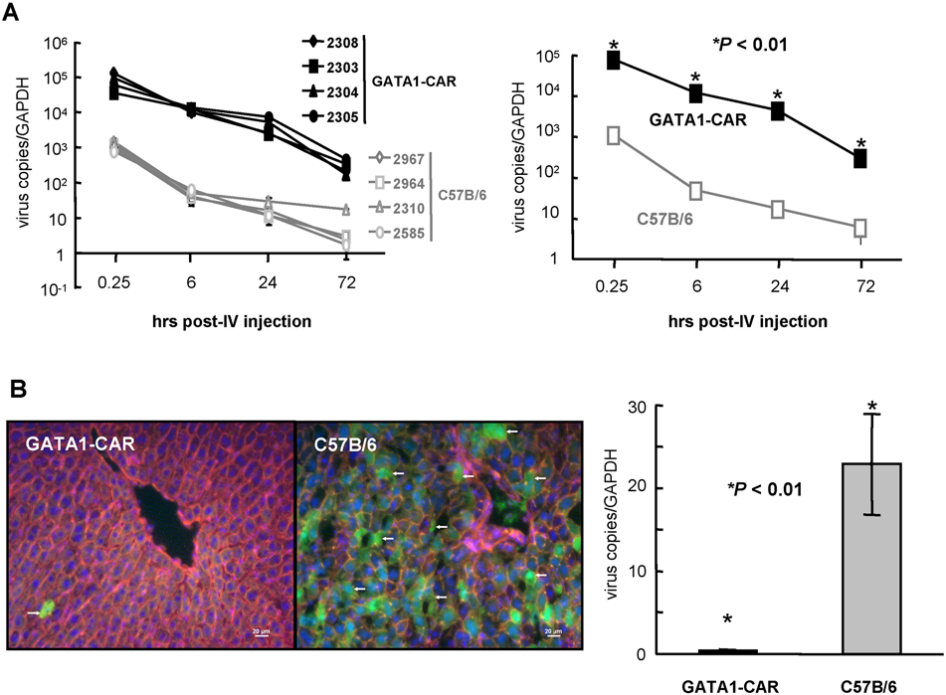
Biodistribution of CAR-tropic HAd5 in GATA1-CAR mice. Mice were injected intravenously with an HAd5 vector expressing GFP (AdGFP) and blood and livers were recovered at the indicated times and assayed for vector genomes and/or GFP expression. A. qPCR of viral genomes in the blood at ∼15 min (0.25 hr), 6 hr, and 1 and 3 days post-injection normalized by copies of GAPDH for each mouse (four GATA1-CAR and four C57B/6 mice, the isogenic strain) (left hand panel) and the mean of each group (right hand panel). qPCR was performed twice in triplicate. B. Histological section from the liver of representative mice at day 3 (72 hr). GFP expression from AdGFP is indicated by the green cells (white arrows). The sections were stained with Hoechst to show the nuclei (in blue) and phalliodin-TRITC to show actin filaments (red, or yellow if an overlap with GFP signal) (left-hand panel). qPCR analysis of the viral genomes in the livers of four GATA1-CAR and four C57BL/6 mice at day 3 (right hand panel). qPCR was performed twice in triplicate.

To better address *in vivo* biodistribution of CAR-tropic Ads, we injected GATA1-CAR and control C57BL/6 mice with a HAd5 vector and quantified viral genome blood half-life and tissue distribution. We found that the viral load in the blood was 1000-fold higher (*P*<0.01) in GATA1-CAR versus isogenic control mice (CAR-negative erythrocytes) during the first 72 hr post-injection (Figure 7A). These data are reminiscent of the studies where human blood cells are routinely positive by qPCR for wild type HAd sequences [6],[7]. In addition, notably absent from the AdGFP-injected GATA1-CAR mice was transgene expression in the liver. Lack of transgene expression was also consistent with the significant (*P*<0.01) ∼25-fold difference in the mean viral load as quantified by qPCR (Figure 7B). The lack of efficient liver infection is also consistent with the generally unexpected and poor infection of rat liver compared to mice and nonhuman primates following injection of CAR-tropic Ad vectors.

Together, these data suggest that CAR expression by rat and human erythrocytes plays a significant role in HAd *in vivo* distribution and, in turn, determining which tissues are susceptible to infection.

## Discussion

We initiated this study to examine a 50-year-old enigma of adenovirus biology. Our results demonstrate that HAd37 interaction with human erythrocytes is primarily due to sialic acid binding via a conserved sialic acid binding site on this subgroup D HAd fiber head. CAV-2, a serotype that like the prototype HAd5 is “CAR-tropic” and by most criteria is unrelated to HAd37, interacts with human erythrocytes via a mechanism depending on several factors, including most notably binding to CAR.

Most subgroup D HAd sialic acid binding site residues are conserved [27], and structural analysis suggest that they bind sialic acid in an equivalent fashion. Unexpectedly, we found that the CAV-2 fiber head harbors a sialic acid binding site. But, in contrast to the well-conserved location of the CAR binding domains on some Ad fiber heads, location of the sialic acid binding site is not conserved.

Following the structure-based identification of the amino acids in the CAV-2 and HAd37 fiber heads that interact with sialic acid, we introduced mutations to assay the role of these sites in sialic acid binding and erythrocyte interaction. In a number of conditions and approaches we showed that hemagglutination by HAd37 depends primarily on sialic acid binding. First, removing sialic acids from erythrocytes with neuraminidase eliminated erythrocyte cross-linking by HAd37 and a chimeric capsid harboring the HAd37 fiber head. Likewise, pre-incubation with neuraminidase significantly reduced hemagglutination caused by protein complexes containing multiple copies HAd37 fiber heads. Second, mutating single residues in the sialic acid binding sites reduced the hemagglutination activity of these protein complexes. Third, wild type HAd37 fiber heads bound more efficiently to erythrocytes or glycophorin than those carrying a point mutation in the sialic acid binding site. Our binding assays using wild type and mutated HAd37 heads also suggested that the sialic acid affinity is partially charge-driven. In addition, although HAd37 head also binds CAR [24], in the context of the virus it does not appear to use CAR as a receptor, possibly due to its relatively short fiber. Therefore, CAR on erythrocytes is likely to be less important for HAd37 biodistribution.

In spite of our structural data showing a well-defined sialic acid binding site, we could not detect notable binding between CAV-2 fiber head and glycophorin, a highly sialated protein. It was possible that the affinity of CAV-2 fiber head to erythrocytes or glycophorin was lower than that of HAd37 fiber head, and that the efficient erythrocyte binding of CAV-2 depended on the avidity of multiple fibers. As the CAV-2 fiber head is less positive than HAd37 fiber head (pI's of 8.4 and 9.2 respectively), this could have explained the lower affinity. The affinity between virus proteins and sialic acid is usually in the millimolar range; therefore it was conceivable that the affinity of single CAV-2 fiber heads to erythrocytes or glycophorin was low. Consistent with this assumption, we previously found that CAV-2 is more neutrally charged than other Ads [32]. Paradoxically though, CAV-2 agglutinates at a lower particle-to-cell ratio than HAd37 and our crystallography data suggested that sialic acid binding should be at least as strong as HAd37. However, one cannot reliably predict affinity from structural data.

The identification of CAR expression by erythrocytes from species that are agglutinated by CAR-tropic Ads is a crucial observation. Using **i)** competition assays with recombinant fiber heads harboring point mutations in the sialic acid and CAR binding sites and **ii)** transgenic mice expressing CAR on erythrocytes, we characterized the unexpected and significant role of CAR in Ad binding.

As mentioned previously, Ad serotypes differ in their ability to bind erythrocytes from various species. Our study suggests that this may be due to **i)** an interaction with different chemical variants, linkages and ratios of sialic acid, **ii)** the presence and affinity to CAR, and/or **iii)** the pI's of the head [33]. For example, some non-CAR-tropic Ads that agglutinate human erythrocytes may preferentially bind Neu5AC, the most abundant sialic acid on human erythrocytes. With respect to charge, both HAd37 and HAd19p bind sialic acid with equal affinity and the only two residues that differ between their heads are not close to the sialic acid-binding site [33]. With the limited amount of data available, subgroup D HAds tend to have heads with higher pI's and interact with sialic acid [33]. Interestingly, the residues that make up the CAV-2 sialic acid binding site are conserved in the CAV-1 fiber head (data not shown), suggesting a conserved sialic acid binding function. With respect to CAR affinity, the CAV-2 fiber head binds human CAR with the highest affinity of any fiber head known [24], which may favor its efficient hemagglutination. In contrast to other Ads, higher temperatures poorly inhibit CAV-2 hemagglutination [12], again suggesting a role for the high-affinity attachment to CAR. This is also consistent with a rather large difference between HAd5 and CAV-2 hemagglutination: to the best of our knowledge the HAd5 head does not harbor a sialic acid binding site and its pI (6.25) is less basic than HAd37 or CAV-2. Finally, we cannot exclude a role of the inter- and intra-species differences in the quantity of CAR expressed by erythrocytes.

Phylogenetically, CAR expression by erythrocytes appears to be random; we tested several other species (dogs, mice, rabbits, lemurs and monkeys) and did not find CAR expression. However, CAR levels on rat erythrocytes were relatively high (Figure 6) and consistent with our hypothesis that CAR-tropic Ads agglutinates human and rat erythrocytes via CAR binding. It is possible, but unlikely, that our lack of detection of CAR on erythrocytes on some species was due to the limit of sensitivity of our bank of anti-CAR antibodies, which nonetheless detected CAR expression on other cell types from these species (not shown). In the context of the significant amount of HAd5-mediated gene transfer data, our data highlights an important parallel. While a 30-gram mouse can be injected intravenously with up to 10^12^ pp of an HAd5 vector with minimal side effects, injection of the same dose in a 250-gram rat is normally lethal [41]. Whether our data have a bearing on the death following portal vein injection of a HAd5 vector during a phase I trial [42] is unknown, but deserves consideration.

Similar to the CAR binding domain in HAd37, we can only speculate about the importance of sialic acid binding in the biology of CAV-2. Although CAV-2 does not agglutinate dog erythrocytes (Figure S4), *Canis lupus familiaris* may not be the original host of CAV-2: seroprevalence against CAV-2 can be found in coyotes, bears, pandas, skunks, mongooses, raccoons and foxes [43]–[45]. It is paradoxical that HAd37 binds CD46, sialic acid and CAR - yet does not use CAR [25] - while CAV-2 binds both sialic acid and CAR and to the best of our knowledge uses only CAR as a receptor [5]. We cannot exclude the possibility that in some cell types sialic acid binding provides a first low-affinity attachment to the cell surface, while CAR-binding is followed in a second step, providing a high-affinity binding. Similar two-step mechanisms have been proposed for other viruses [46]. It is tempting to speculate that sialic acid binding may play a role in the preferential transduction of neurons by CAV-2 vectors [47]–[50]. Our crystal structure of CAV-2 fiber head in complex with CAR and sialyl-D-lactose demonstrates that the ternary complex of the three molecules is stable. There is no indication that this should not be the case also *in vivo*.

Our data and numerous reports describing the *ex vivo* interaction of CAR-binding HAd5 with human/rat erythrocytes [12], [21]–[23], [29], [36], [51]–[53] strongly suggest this interaction probably occurs after the intravascular injection of CAR-tropic vectors. Notably, Lyons et al. showed that that >90% HAd5 vector DNA was associated with blood cells following intratumoral injection during a clinical trial [34].

It is likely that erythrocyte binding occurs during wild type infection of some HAds (as well as coxsackie B viruses) that use CAR, which will certainly lead to altered biodistribution. HAd DNA is routinely found in human blood cells by PCR. It is also a common misconception that Ads are rapidly cleared following a classical immune response. Numerous clinical cases strongly suggest that latent HAds can readily resurface if the host is immunosuppressed. The fate of particles that stick to erythrocytes under natural or artificial (i.e. vector injections) conditions is probably complex. For example, HAd5-induced liver disease in immunocompromised humans is relatively common. In contrast, immunocompromised nonhuman primates are rarely diagnosed with simian Ad (SAV)-induced liver disease [54]. Does CAR expression on erythrocytes lead to an advantage for host or virus? Has CAR expression on erythrocytes put a selective pressure on Ads (and coxsackie B viruses that also bind CAR) to avoid an erythrocyte virus trap [28]? Or has CAR expression by erythrocytes allowed HAds to thrive because it has allowed open access to so many more tissues and cell types?

In summary, our results resolve a longstanding enigma of Ad-erythrocyte interaction *in vitro* and *in vivo.* In addition, we provide new insights into virus-erythrocyte interactions that will allow us to better understand HAd pathogenesis and facilitate the engineering of safer, more efficient gene transfer vectors. Although in most *in vivo* scenarios hemagglutination *per se* is unlikely to occur due to the turbulence that erythrocytes encounter in the circulation, sequestering of vector particles by erythrocytes must diminish gene transfer efficacy. Interest in Ad biology is continually growing due to the increasing incidence of HAd-induced morbidity and mortality during immunosuppression [55],[56], and because Ad-derived vectors are the most commonly used vectors in gene therapy clinical trials. The identification of fiber head mutants that do not bind human erythrocytes may be of interest. Equally important may be the need to screen fiber heads from other serotypes for sialic acid and CAR binding domains. Combined with HAd interactions with vitamin K-dependent coagulation factors [39], our study adds another critical element in the interaction with blood components, biodistribution and pathogenesis.

## Materials and Methods

### Viruses, vectors and antibodies

CAV-2 (CAVGFP), HAd5 (AdGFP), HAd37, and the hybrid HAd5-CAV-2H (Ad5Luc1-CK) and HAd5-HAd37F (Ad37f) vectors were prepared as previously described [30],[31],[57],[58]. Briefly, CAVGFP and AdGFP are E1-deleted vectors expressing GFP. The capsids contain no modifications. The hybrid HAd5-CAV-2H vector contains the fiber head of CAV-2 on the HAd5 capsid. HAd5-HAd37F vector contains the HAd37 fiber (shaft and head) on the HAd5 capsid. All vectors were purified by double banding on CsCl gradients, CsCl was removed using PD-10 columns (Pharmacia). The vectors were stored in phosphate-buffered saline (PBS) containing 10% glycerol at −80°C. Stock titers were >1×10^12^ physical particles/ml with >1 infectious particle/5 physical particles. Anti-CAR antibodies tested in this study included E1.1 a monoclonal mouse (S. Hemmi, University of Zurich), CAR1605 polyclonal rabbit (J. Zabner), MoAbE(mh)1 monoclonal mouse (S. Carson, University of Nebraska), and AF2654 (anti-mouse) and AF3336 (anti-human) polyclonal goat (R & D Systems).

### Negative staining

The recombinant fiber heads and dodecahedral sample were loaded between the mica-carbon interface as described [59]. The samples were stained using 2% sodium silico tungstate pH 7.5 and air-dried. Images were taken under low-dose conditions in an EX1200-II JEOL electron microscope working at 100 kV and with a nominal magnification of 40,000. The images were scanned on a Z/I Imaging scanner (Photoscan TD) with a pixel size of 14 mm (3.5 Å per pixel at the sample level).

### Generation of recombinant fiber head and labeling with fluorophores

Fiber head constructs were cloned into pPROEX HTb (Life Technologies) and expressed with a cleavable His-tag as described previously [24]. HAd37 fiber head constructs contain residues 177–365 and CAV-2 fiber head constructs contain residues 358–542. Point mutations were introduced using the QuikChange Site-Directed Mutagenesis Kit (Stratagene) and polymerase chain reaction (PCR). Protein purification was performed as described [24]. Briefly, cells were incubated in lysis buffer (20 mM Tris-HCl pH 7.5, 300 mM NaCl, 20 mM imidazole, (Boehringer Complete EDTA-free protease inhibitor cocktail), centrifuged, and fiber head bound to a Ni-NTA column (Qiagen). Eluted protein was either directly loaded onto a Superdex200 column for hemagglutination experiments with tagged fiber head, or incubated overnight with 1/100 His-tagged tobacco etch virus (TEV) protease at 10°C. Proteins were then dialyzed against lysis buffer and uncleaved protein and TEV protease bound to a Ni-NTA resin. Untagged fiber head was loaded onto a Superdex200 column using the same buffer as for tagged protein (20 mM Tris pH7.5 and 300 mM NaCl). CAV-2H^CAR−^ was labeled using Alexa488 Microscale Protein Labeling Kit (Molecular Probes). CAV-2H^wt^ was dialyzed in PBS 0.1 M NaCO_3_ pH 9.3 and labeled using mono-reactive dyes (Cy3, Cy5 or Alex488, Amersham Bioscience) for 45 min at room temperature. The elution of labeled protein was performed with 2 ml of PBS using NAP5 column (GE Healthcare) pre-equilibrated with 10 ml PBS. The final dye/protein ratios (∼2.4 for each) were determined using NanoDrop ND-100 spectrophotometer.

### Crystallization of CAV-2 fiber head in complex with sialyl-lactose

Untagged CAV-2 fiber head was concentrated to 17 mg/ml in crystallization buffer (150 mM NaCl, 20 mM Tris pH 7.5, 8 mM sialyl-D-lactose) and crystallized in hanging drops containing 1 µl protein solution and 1 µl well solution (5% PEG 4000, 5% isopropanol, 0.1 M HEPES pH 7.4). Single crystals were transferred and frozen in a cryoprotectant solution (65% well solution, 25% glycerol, approximately 80 mM sialyl-D-lactose). The sialyl-D-lactose (Sigma) corresponds to α2-3 N-acetylneuraminosyl-D-lactose (according to the supplier).

### Purification and crystallization of fiber head complexes containing CAR D1 and sialyl-D-lactose

The cloning, expression and crystallization of HAd37 and CAV-2 fiber head CAR D1 complexes (space groups I23 and I422) was performed as described previously [24]. After crystal growth, sialyl-D-lactose (Sigma) was added to the drop to a final concentration of 10 to 50 mM. Crystals were frozen the next day without adding cryoprotectant solution to crystals with CAV-2 fiber head complex, and with 20% glycerol in the crystallization condition for crystals with HAd37 fiber head complex.

### Data collection and processing

Details of the data collection and refinement of the three structures (CAV-2+sialyl-lactose, CAV-2+CAR D1+sialyl-lactose and HAd37+CAR D1+sialyl-lactose) are given in Table S1. All crystallographic data were collected at the European Synchrotron Radiation Facility (ESRF) and processed with XDS [60]. All structures were solved by molecular replacement using PHASER [61] and refined with REFMAC [62]. COOT [63] was used for the visualization of all models and electron density maps, and for the superposition of different models. PROCHECK [64] and MolProbity [65] were used for the validation of the obtained models. Representations of the protein models and the electron density were made with PYMOL [66] and GRASP [67]. Binding interfaces were visualized with DIMPLOT [68]. The structure-based sequence alignment was made with SARF, modified manually in SEAVIEW [69] and visualized with ESPript [70]. The CAV-2+sialyl-lactose structure contains two fiber head trimers in the asymmetric unit. The CAV-2+CAR D1+sialyl-lactose contains four trimeric fiber head - CAR D1 complexes in the asymmetric unit. Due to the modest resolution of this structure, NCS restraints and TLS refinement was used. The HAd37+CAR D1+sialyl-lactose structure has one fiber head monomer with bound CAR D1 in the asymmetric unit.

### Cloning and purification of HAd3 penton dodecahedra in complex with chimeric mini-fibers

Chimeric mini-fiber constructs were cloned by PCR in two steps. First, DNA fragments were produced using as template HAd3 genomic DNA (for fragment 1) or pPROEX HTb vectors coding for wild type or mutant HAd37 fiber head (for fragments 2 and 3). Fragment 1 coded for HAd3 tail and shaft motives 1+2 (primers were AAT AAT CCA TGG CCA AGC GAG CTC GG and GTT CCA TGC TAC CAA GGA TCC ATC AGT AG). Fragments 2 and 3 coded for wild type and mutant (K345E) HAd37 fiber head (primers used were CTA CTG ATG GAT CCT TGG TAG CAT GGA AC and AAT AAT GAA TTC TCA TTC TTG GGC AAT ATA GG). In a second PCR reaction, fragment 1 was annealed to fragment 2 or 3 using primers AAT AAT GAA TTC TCA TTC TTG GGC AAT ATA GG and AAT AAT CCA TGG CCA AGC GAG CTC GG. The resulting longer fragments coded for complete mini-fiber containing HAd3 tail+shaft and either wild type or mutant (K345E) HAd37 fiber head. These were cloned into pPROEX HTb, and expressed at 25°C in E. coli strain BL21 Star (DE3) (Life Technologies) together with an N-terminal cleavable 6×His-tag. Cells were re-suspended in lysis buffer and sonicated. The cell lysate was centrifuged for 30 min at 25000g and the supernatant loaded on a Ni-NTA column (Qiagen). Protein bound to the resin was washed with 20 mM Tris-HCl pH 7.5, 300 mM NaCl, 50 mM imidazole and eluted in 20 mM Tris-HCl pH 7.5, 150 mM NaCl, 500 mM imidazole. To remove the His-tag, mini-fibers were incubated overnight with 1/100 His-tagged TEV protease at 10°C. Imidazole was removed by dialysis against lysis buffer. Uncleaved protein and TEV protease were removed by binding to a Ni-NTA resin. HAd3 penton forms multimers (dodecahedra) consisting of twelve penton bases each [9],[71]. P. Fender (Institut de Biologie Structurale, Grenoble) generously supplied purified HAd3 dodecahedra. Mini-fibers and dodecahedra were mixed at an approximate molecular ratio of 1:10 and incubated at 4°C overnight. The mixture was loaded onto a Superdex200 column (Amersham) to remove excess fiber. The buffer contained 20 mM Tris pH7.5 and 300 mM NaCl. Dodecahedra in complex with mini-fiber were eluted with the void volume and were visualized with an electron microscope. All mini-fiber constructs carrying HAd3 tail+shaft and a CAV-2 fiber head were unstable, possibly because they were misfolded, or they did not bind to HAd3 dodecahedra.

### Flow cytometry

Non-treated, mock-treated or neuraminidase-treated erythrocytes and non-tagged fiber heads were incubated for 20 min at 4°C. Cell samples serving as negative control were incubated with PBS instead of fiber head solution. The cells were pelleted at 800g, re-suspended in PBS containing 1:100 rabbit anti-HAd37 fiber head serum (gift from N. Arnberg, University of Umeå) or 1:100 purified rabbit antibodies against CAV-2 fiber head, and incubated for 20 min at 4°C. The cells were pelleted as before, re-suspended in PBS containing 1:100 anti-rabbit FITC labeled antibody (Sigma) and incubated in the dark at 4°C for 20 min. The cells were pelleted again, re-suspended in PBS, and injected into a FACSCalibur apparatus (BD Biosciences). The results were analyzed using CellQuest and FlowJo software (BD Biosciences).

### Hemagglutination assays

Human, rat (Wistar), dog (beagle), vervet (Chlorocebus *pygerythrus*), cynomologous monkey, rhesus macaque, guinea pig, rabbit (New Zealand White), mouse (C57BL/6) or GATA1-CAR [28] mouse blood was collected in EDTA, heparin or Alsever's solution. Erythrocytes were purified using Ficol gradients, washed twice in PBS EDTA (5 min at 2000 rpm) and stored less than 48 hrs in PBS containing 5 mM EDTA. For the human and GATA1-CAR mouse erythrocytes two fractions of packed erythrocytes were re-suspended separately in neuraminidase buffer each. Neuraminidase α(2-3,-6,-8 and-9) from *Arthrobacter ureafaciens* (Sigma) or α(2-3,-6 and -8) (Ozyme) was activated as recommended, and added to one of the two erythrocytes fractions. Both mock- (without neuraminidase) and neuraminidase-containing fractions were incubated for 1 hr at 37°C. The cells were then washed 5 times with PBS to remove neuraminidase and buffer, and re-suspended in PBS containing 5 mM EDTA. Virus, dodecahedra ± chimeric mini-fibers and CAV-2 fiber head multimers were diluted with PBS in 10-fold (in the first column) and then 2-fold (horizontal) steps and added to 96-well plates with cone-shaped bottoms. An equal number of purified erythrocytes (either non-treated, neuraminidase-treated, or mock-treated) was added to each well and incubated at room temperature or 37°C for at least 3 hrs. The blocking protein (∼1 µg/well) was incubated with erythrocytes for 1 h at 4°C with slow rotation. All assays were repeated at least 3 times.

### Sedimentation with erythrocytes

In a volume of 100 µl, CAVGFP or AdGFP (1 particle/cell) was incubated with 2.5×10^7^ mock-treated or with neuraminidase-treated erythrocytes with PBS, or PBS supplemented with NaCl to bring its final concentration to 225 or 400 mM. The samples were incubated at room temperature for 15 min, and then centrifuged for 5 min at 5000 rpm in a microfuge. Aliquots of the supernatant were removed and incubated with 1×10^5^ cells in 12-well plates. The cells were trypsinized and assayed for GFP expression by flow cytometry 24 hrs post-incubation. Data were analyzed using CellQuest. The assays were performed twice and in quadruplicate.

### Western blot

Control cells and tissues were lysed with 100 µl SDS buffer (10^6^ cells) and benzonase for 1 h at 37°C. The liver, peripheral blood mononuclear cells, NIH 3T3 cells (mouse fibroblasts) and 293 cell (human embryonic kidney) extracts (40 mg/ml) were resuspended in 100 µl of SDS-sample buffer. Erythrocytes (∼200 µl) were lysed in ddH_2_O, the membranes were pellet for 10 min at 14,000 RPM in a microfuge and resuspended in 100 µl of SDS-sample buffer. SDS-PAGE was performed using a 5% acrylamide/bis-acrylamide stacking gel and a 12% acrylamide/bis-acrylamide running gel. Membranes were blocked with TBS-Tween, 10% milk at room temperature. The rabbit anti-CAR antibody CAR1605 was diluted 2000-fold for 1 h at RT in TBS-Tween 10% milk. The secondary anti-rabbit antibody was used at a dilution of 1/5000 for 30 min at room temperature in TBS-Tween 10% milk.

### *In vivo* injection of Ad vector

Four adult GATA1-CAR and four control C57BL/6 mice were injected with 1.2×10^11^ pp of AdGFP via the tail vein. Blood (∼100 µl) was taken by tail vein bleeds at 0.25, 6, 24 and 72 hr. The mice were sacrificed at 72 hr by lethal injection, and the organs were perfused with PBS via cardiac puncture. The liver, lung and spleen were recovered, divided into parts for qPCR or histology. The organs used for histology were fixed in 4% PFA for 24 hr then placed in 20% sucrose for 24 hr, and embedded in OCT matrix (CellPath, Powys, UK). Sections (10-µm-thick) were stained with 0.2 µg/ml bisBenzimide Hoechst (Sigma-Aldrich) and 1 ng/ml phalloidin-TRITC (Sigma-Aldrich) before being mounted. Images were acquired using a Zeiss microscope and processes using the MetaMorph (Molecular Devices, Wokingham, UK).

### Ethics

The experimental protocols involving animals were approved by the University of Massachusetts Medical School Institutional Animal Care and Use Committee.

### Quantitative PCR

Total DNA from blood and liver were extracted by using the High Pure DNA Isolation kit (Roche Diagnostics). qPCR was performed with a Light Cycler (Roche Diagnostics) using the Platinum Taq DNA polymerase (Invitrogen) and SYBR Green qPCR master mix [72]. The primer pairs used for GAPDH were: GAPDH forward, 5′ ACA GTC CAT GCC ATC ACT GCC 3′; GAPDH reverse, 5′ GCC TGC TTC ACC ACC TTC TTG 3′; and the EGFP: forward, 5′ CAG AAG AAC GGC ATC AAG GT 3′; eGFP reverse, 5′ CTG GGT GCT CAG GTA GTG G 3′. Data are expressed as a ratio of GAPDH to EGFP.

### Statistical analysis

Data were analyzed using a one-way ANOVA and *post-hoc* comparisons were made using an unpaired Student's *t*-test.

## Supporting Information

Figure S1 Electron micrographs of dodecahedra ± fiber. A) Ad3 penton dodecahedra B) Ad3 penton dodecahedra in complex with chimeric minifiber containing wild type HAd37 fiber head. To the right of the photos are computer generated models of the structures. Below are enlarged images of the dodecahedra ± fiber.
(1.11 MB TIF)

Figure S2 Binding of wild type and mutant fiber heads to human erythrocytes. A) SPR curves obtained with different concentrations of wild type HAd37 fiber head (HAd37Hwt) and immobilized glycophorin (GP). B) SPR curves obtained with HAd37Hwt and mutant HAd37 fiber heads (HAd37HSA-1 and HAd37HSA-2) at 2 µM and immobilized GP. C) SPR curves obtained with HAd37Hwt and mutant HAd37 fiber heads at 2 µM and immobilized asialoGP.
(0.27 MB TIF)

Figure S3 Sequence of the CAV-2 and HAd37 fiber heads. Structure based sequence alignment of HAd37 and CAV-2 fiber heads. Residues forming hydrogen bonds with sialic acid are highlighted with a blue background; residues involved in hydrophobic contacts with sialic acid are highlighted with yellow background; residues providing both types of contacts are highlighted in blue and yellow.
(0.99 MB TIF)

Figure S4 Flow chart showing competition-based agglutination assays.
(1.87 MB TIF)

Figure S5 Relative agglutination with erythrocytes from different species.
(0.10 MB TIF)

Table S1 Summary of data collection and refinement statistics.
(0.09 MB DOC)
