# Supplementary figures and images for: The Cell Adhesion Molecule “CAR” and Sialic Acid on Human Erythrocytes Influence Adenovirus In Vivo Biodistribution
Source: PLoS Pathog. 2009 Jan 2;5(1):e1000277. doi: 10.1371/journal.ppat.1000277 (PMC2607015; doi:10.1371/journal.ppat.1000277)

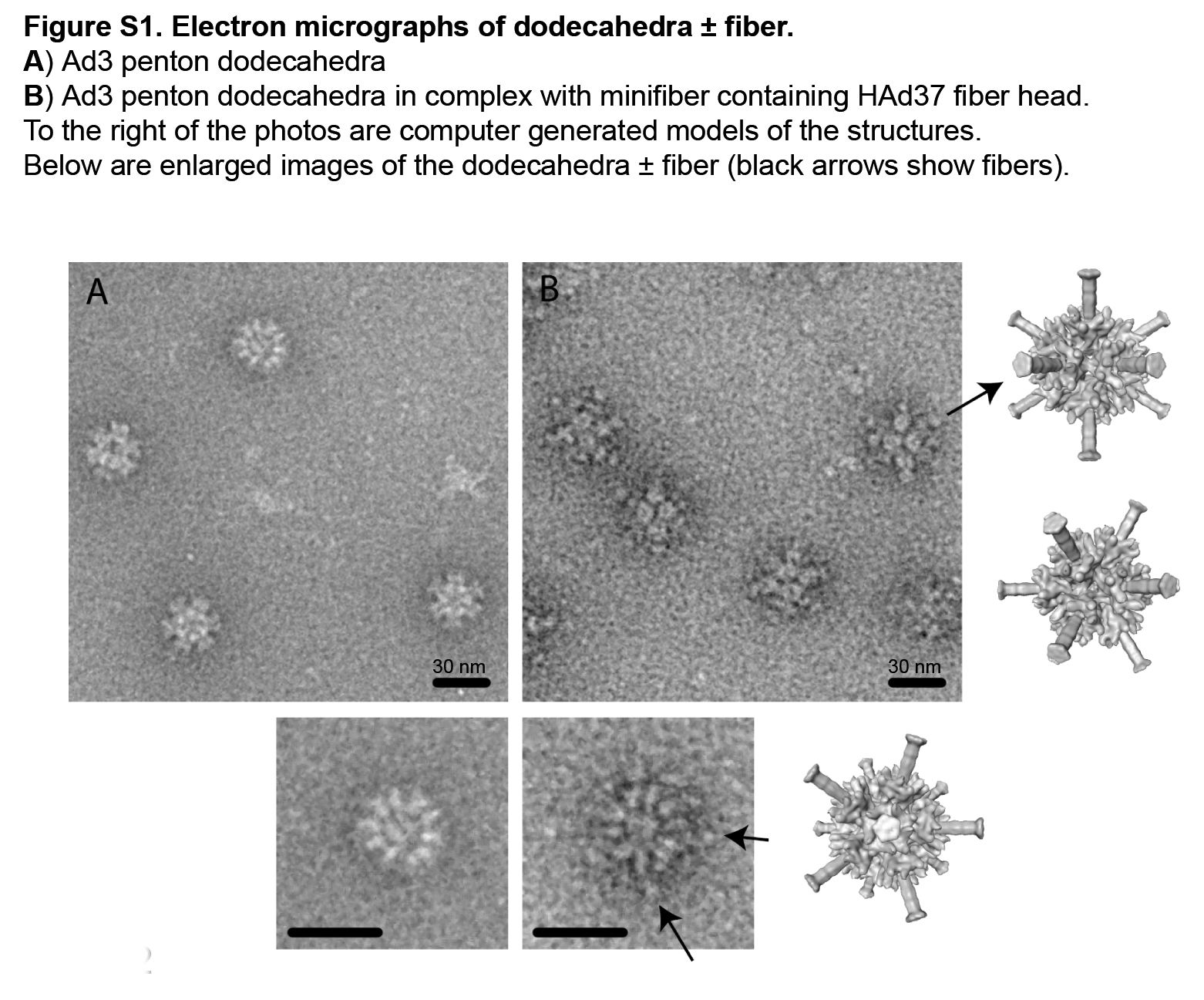

Supplement: Figure S1 — Electron micrographs of dodecahedra ± fiber. A) Ad3 penton dodecahedra B) Ad3 penton dodecahedra in complex with chimeric minifiber containing wild type HAd37 fiber head. To the right of the photos are computer generated models of the structures. Below are enlarged images of the dodecahedra ± fiber. (1.11 MB TIF) [file ppat.1000277.s001.tif]

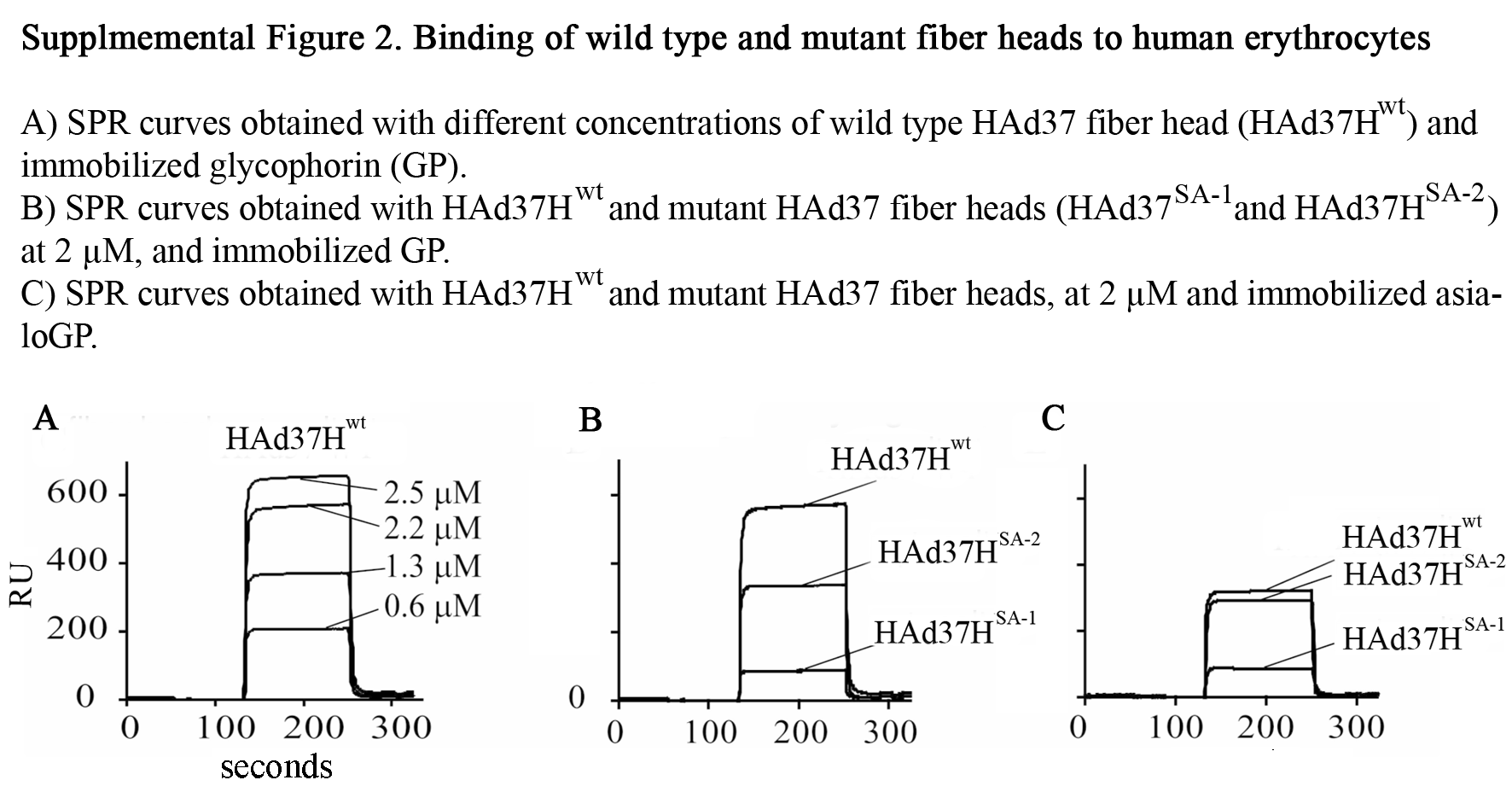

Supplement: Figure S2 — Binding of wild type and mutant fiber heads to human erythrocytes. A) SPR curves obtained with different concentrations of wild type HAd37 fiber head (HAd37Hwt) and immobilized glycophorin (GP). B) SPR curves obtained with HAd37Hwt and mutant HAd37 fiber heads (HAd37HSA-1 and HAd37HSA-2) at 2 µM and immobilized GP. C) SPR curves obtained with HAd37Hwt and mutant HAd37 fiber heads at 2 µM and immobilized asialoGP. (0.27 MB TIF) [file ppat.1000277.s002.tif]

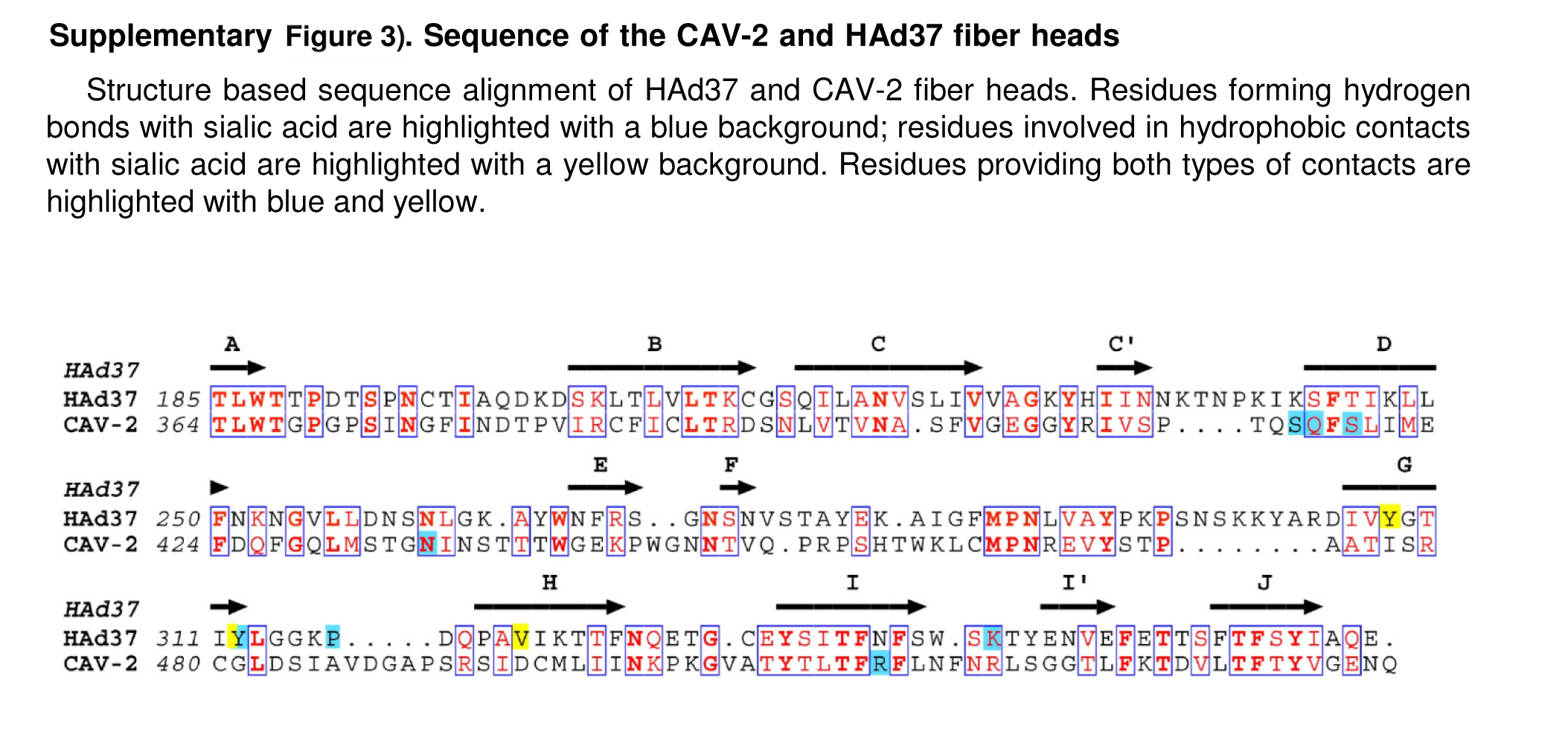

Supplement: Figure S3 — Sequence of the CAV-2 and HAd37 fiber heads. Structure based sequence alignment of HAd37 and CAV-2 fiber heads. Residues forming hydrogen bonds with sialic acid are highlighted with a blue background; residues involved in hydrophobic contacts with sialic acid are highlighted with yellow background; residues providing both types of contacts are highlighted in blue and yellow. (0.99 MB TIF) [file ppat.1000277.s003.tif]

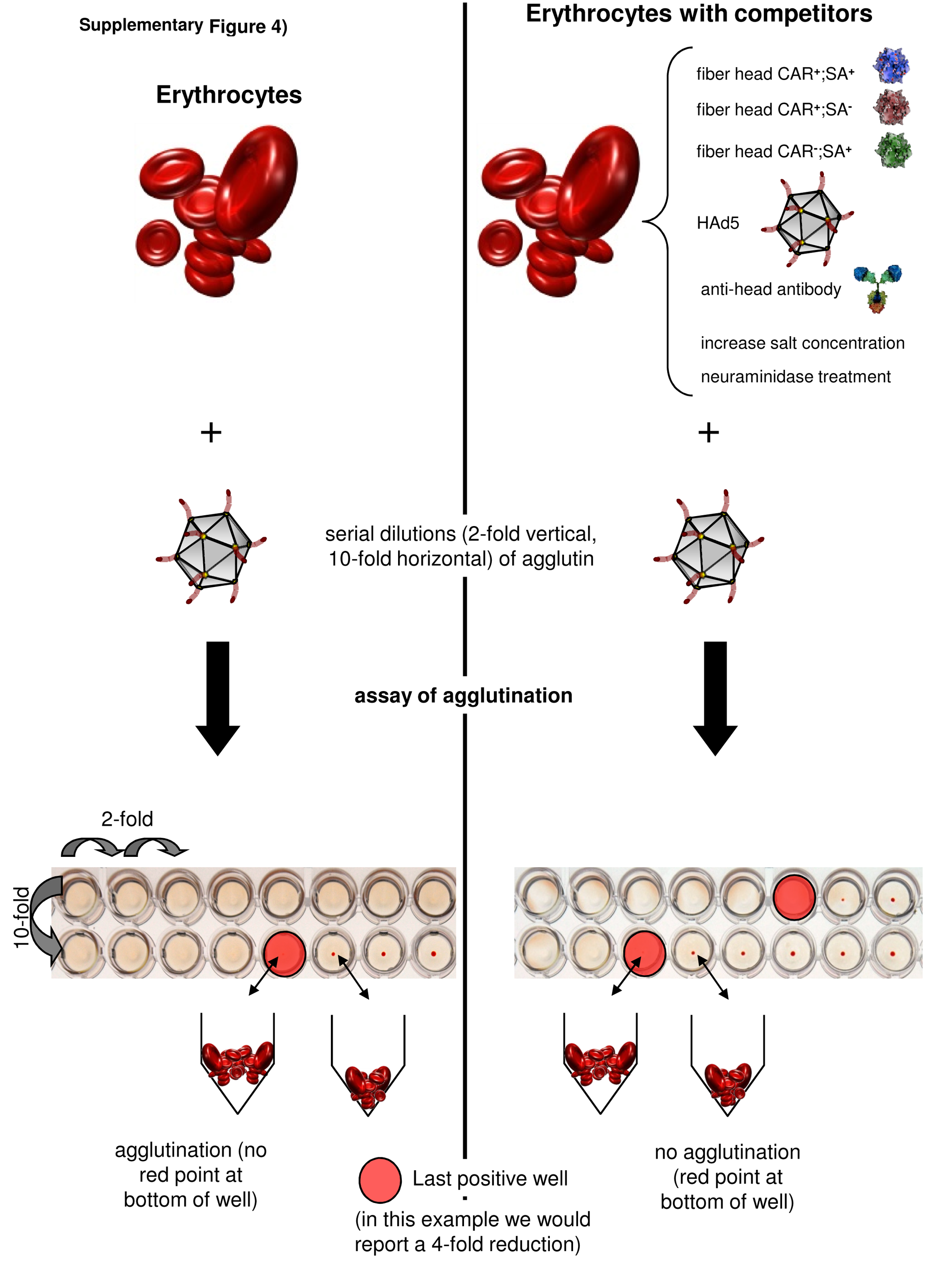

Supplement: Figure S4 — Flow chart showing competition-based agglutination assays. (1.87 MB TIF) [file ppat.1000277.s004.tif]

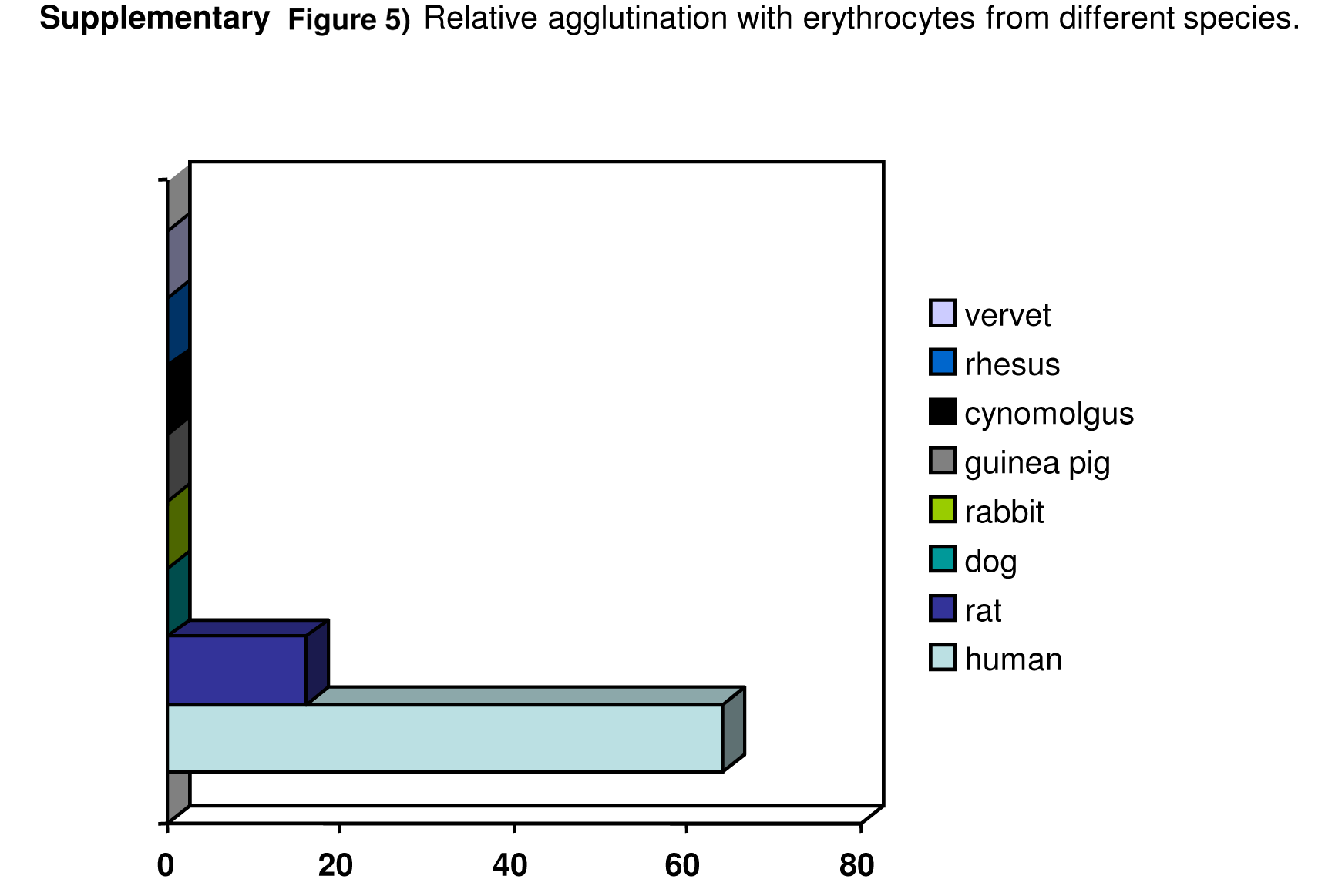

Supplement: Figure S5 — Relative agglutination with erythrocytes from different species. (0.10 MB TIF) [file ppat.1000277.s005.tif]
