# Supplementary material for: The Cell Adhesion Molecule “CAR” and Sialic Acid on Human Erythrocytes Influence Adenovirus In Vivo Biodistribution
Source: PLoS Pathog. 2009 Jan 2;5(1):e1000277. doi: 10.1371/journal.ppat.1000277 (PMC2607015; doi:10.1371/journal.ppat.1000277)
Supplement: Table S1 — Summary of data collection and refinement statistics. (0.09 MB DOC) [file ppat.1000277.s006.doc]

Table S1: Summary of Data Collection and Refinement Statistics.

*Indicates highest resolution shell.

|  | CAV-2 fiber head with sialyl-lactose | CAV-2 fiber head with CAR D1 and sialyl-lactose | HAd37 fiber head with CAR D1 and sialyl-lactose |
| --- | --- | --- | --- |
| **Data Collection** |  |  |  |
| Beamline (ESRF) | ID29 | ID14-EH4 | ID14-EH4 |
| Wavelength (Ǻ) | 0.976 | 0.939 | 0.939 |
| Space Group | *C*2 | *I*422 | *I*23 |
| Cell Dimensions | a = 215.0 Ǻ b = 61.3 Ǻ  c = 143.1 Ǻ α = γ = 90˚  β = 131.7˚ | a = b = 221.15 Ǻ  c = 391.6 Ǻ  α = β = γ = 90˚ | a = b = c = 132 Ǻ  α = β = γ = 90˚ |
| Resolution Range (Ǻ) | 46.1 - 1.90 (1.90-1.97)* | 50 - 2.9 (2.9-3.0)* | 50 - 1.55 (1.55-1.58)* |
| Completeness (%) | 99.4 (98.8)* | 98.3 (93.2)* | 99.9 (99.9)* |
| R-merge | 0.126 (0.424)* | 0.146 (0.593)* | 6.8 (56.7)* |
| I/σI | 10.2 (4.1)* | 14.2 (3.3)* | 22.7 (4.9)* |
| Unique reflections | 109731 ( 11113)* | 105112 (9515)* | 55340 (3069)* |
| Redundancy | 3.71 (3.63)* | 8.2 (8.4)* | 11.0 (10.9)* |
| **Refinement** |  |  |  |
| Resolution Range (Ǻ) | 46.1 - 1.90 (1.90-1.95)* | 50 - 2.90 (2.90-2.98)* | 50 - 1.55 (1.55-1.59)* |
|  |  |  |  |
| No. of reflections used in refinement | 104251 | 104076 | 52533 |
| No. of free reflections | 5480 | 1034 | 2807 |
|  |  |  |  |
| R-factor | 0.157 (0.194)* | 0.191 (0.282)* | 0.171 (0.218)* |
| R-free | 0.195 (0.258)* | 0.247 (0.341)* | 0.176 (0. 214)* |
| Total no. of atoms (non-hydrogen) | 9900 | 28671 | 2830 |
| No. water molecules | 1152 (+ 6 glycerol) | 158 | 400 |
| Solvent content (%) | 58 | 56 | 53 |
| Mean B-value (Ǻ^2^) | 9.2 | 31.2 | 16.6 |
| **Ramachandran plot of non-glycine and non-proline residues** : |  |  |  |
| Most favorable regions (%) | 88.8 | 85.4 | 86.1 |
| Additional allowed (%) | 11.2 | 14.4 | 13.5 |
| Generously allowed (No.) | - | 5 | - |
| Disallowed regions (No.) | - | - | 1 (Thr189) |
| **RMS deviations from ideal values** |  |  |  |
| Bond distances (Ǻ) | 0.008 | 0.010 | 0.007 |
| Angles (°) | 1.27 | 1.36 | 1.14 |
